# ARE OLDER ADULTS’ SOCIAL PARTICIPATION AND RELATIONSHIP QUALITY RELATED TO THEIR SPOUSE’S COGNITION?

**DOI:** 10.1093/geroni/igac059.3006

**Published:** 2022-12-20

**Authors:** Changmin Peng, Yan Lin, Shan Qu, Yiyang Yuan

**Affiliations:** University of Massachusetts Boston, Brookline, Massachusetts, United States; University of Massachusetts Boston, Bedford, New Hampshire, United States; University of Massachusetts Boston, Boston, Massachusetts, United States; University of Massachusetts Chan Medical School, Worcester, Massachusetts, United States

## Abstract

Limited studies have quantified older adults’ profiles of social participation and relationship quality. How such profiles were associated with their spouse/partner’s cognitive function also remains unknown. Using the Health and Retirement Study (2014/2016), we identified 3,722 community-dwelling, cognitively intact, married/partnered respondents living with their spouse/partner. Spouse/partner’s cognitive function was ascertained by the Langa-Weier Classification [Intact/Cognitively Impaired but not Demented (CIND)/Demented]. Social participation was measured by the frequency of volunteer, charity, education, sport/social clubs, and non-religious organization activities. Relationship quality was measured by the perceived positive and negative support from spouse, children, relatives, and friends. Latent profile analysis identified profiles of social participation and relationship quality. Multinomial logistic regression estimated the association between spouse/partner’s cognitive function and the respective profiles. Three social participation profiles were identified: (1) Limited social participation (prevalence: 69%; reference); (2) Frequent volunteer participation (10%); (3) Frequent non-volunteer participation (21%). Three relationship quality profiles were identified: (1) Positive overall support (68%; reference); (2) Positive spousal support (18%); (3) Negative spousal support with positive non-spousal support (14%). Those with a spouse/partner that were CIND or demented were significantly less likely to frequently participate in volunteer or non-volunteer activities, while more likely to perceive negative spousal support and positive non-spousal support. Given these findings and the essential role of social participation and relationship quality in older adults’ well-being, programs focusing on older adults living with a spouse/partner with impaired cognition are needed to help them maintain social connectedness.

